# Ontology-based venous thromboembolism risk assessment model developing from medical records

**DOI:** 10.1186/s12911-019-0856-2

**Published:** 2019-08-08

**Authors:** Yuqing Yang, Xin Wang, Yu Huang, Ning Chen, Juhong Shi, Ting Chen

**Affiliations:** 1 Department of Computer Science and Technology, Institute for Artificial Intelligence, State Key Lab of Intelligent Technology and Systems, Tsinghua University, Beijing, 100084 China; 2 Tsinghua-Fuzhou Institute of Digital Technology, Beijing National Research Center for Information Science and Technology, Tsinghua University, Beijing, 100084 China; 3 Department of Ultrasound, Peking Union Medical College Hospital, Beijing, China; 4 Peking Union Medical College, Beijing, China; 5 Department of Respiration, Peking Union Medical College Hospital, Beijing, China

**Keywords:** Medical record, Venous thromboembolism (VTE), Natural language processing (NLP), Risk assessment, Machine learning (ML)

## Abstract

**Background:**

Padua linear model is widely used for the risk assessment of venous thromboembolism (VTE), a common but preventable complication for inpatients. However, genetic and environmental differences between Western and Chinese population limit the validity of Padua model in Chinese patients. Medical records which contain rich information about disease progression, are useful in mining new risk factors related to Chinese VTE patients. Furthermore, machine learning (ML) methods provide new opportunities to build precise risk prediction model by automatic selection of risk factors based on original medical records.

**Methods:**

Medical records of 3,106 inpatients including 224 VTE patients were collected and various types of ontologies were integrated to parse unstructured text. A workflow of ontology-based VTE risk prediction model, that combines natural language processing (NLP) and machine learning (ML) technologies, was proposed. Firstly ontology terms were extracted from medical records, then sorted according to their calculated weights. Next importance of each term in the unit of section was evaluated and finally a ML model was built based on a subset of terms. Four ML methods were tested, and the best model was decided by comparing area under the receiver operating characteristic curve (AUROC).

**Results:**

Medical records were first split into different sections and subsequently, terms from each section were sorted by their weights calculated by multiple types of information. Greedy selection algorithm was used to obtain significant sections and terms. Top terms in each section were selected to construct patients’ distributed representations by word embedding technique. Using top 300 terms of two important sections, namely the ‘Progress Note’ section and ‘Admitting Diagnosis’ section, the model showed relatively better predictive performance. Then ML model which utilizes a subset of terms from two sections, about 110 terms, achieved the best AUC score, of 0.973 ± 0.006, which was significantly better compared to the Padua’s performance of 0.791 ± 0.022. Terms found by the model showed their potential to help clinicians explore new risk factors.

**Conclusions:**

In this study, a new VTE risk assessment model based on ontologies extraction from raw medical records is developed and its performance is verified on real clinical dataset. Results of selected terms can help clinicians to discover meaningful risk factors.

## Background

As a common complication for inpatients, venous thromboembolism (VTE) comprising pulmonary embolism (PE) and deep venous thrombosis (DVT) is a preventable cause of death. American guidelines has reported that hospital admission is related to nearly 25% of all VTE patients and 10% deaths of inpatients is caused by PE [1]. Prophylaxis against VTE can reduce mortality efficiently. However, the pathogenesis of VTE is complex. Although VTE has shown associations with many risk factors such as elder age, obesity, cancer, immobility and inflammatory bowel disease [2, 3], there isn’t an universal VTE risk assessment method. American College of Chest Physicians recommends the Padua model, but its performances was poor when applied to Chinese inpatients in the Internal Department [4]. VTE is closely related to ethnic background and disease spectrum. However, Chinese differed from western population in the disease risk assessment. Thus, it is essential to find the potential VTE risk factor and develop prediction model specifically for Chinese inpatients.

Padua model is a traditional linear weighting approach, which was proposed by a prospective cohort study in the hospital in Italy. Risk factors include 11 features and are assigned different weights shown in Table 1. Four factors, including active malignant cancer/chemotherapy, previous VTE, reduced mobility and thrombophilic condition have three points. The weight of ‘recent trauma/surgery’ is two points and the rest of the factors are one point. During the assessment of a patient’s VTE risk, points of patients’ risk factors are summed up. The patient is classified as high risk when the Padua score ≥ 4. It is trivial that the number of risk factors used by Padua model is relatively limited. Some variables, such as blood transfusion and mechanical ventilation, which showed obvious difference between patients with or without VTE in Chinese hospital, are not considered [5]. Analyzing Chinese VTE patients’ medical records in detail may uncover new variables associated with VTE and improve Padua model.

**Table 1:** Padua risk assessment model

Risk Factors	Score
Active malignant cancer/chemotherapy	3
Previous VTE	3
Reduced mobility	3
Thrombophilic condition	3
Recent trauma/surgery	2
Age > =70	1
Heart/respiratory failure	1
Acute myocardial infarction/ischemic stroke	1
Acute Infection/rheumatologic disorder	1
BMI > =30 kg/m^2^	1
Ongoing glucocorticoid treatment	1

The Padua score ≥ 4 is classified as high risk

The rapid development of medical informatization and electronic health record (EHR) system allows the accumulation of increasing number of medical records. This provides the possibility of investigating diseases in more elaborate and precise methods, compared to traditional approaches with small sample size and limited variables. Many researchers have studied relationships between various diseases and risk factors using machine learning (ML) and natural language processing (NLP) methods which showed promising results. SF Weng, et al. [6] compared predictive validities of multiple ML methods on cardiovascular risk assessment, R Casanova, et al. [7] analyzed the Alzheimer’s disease risk by regularized logistic regression, and P Ferroni, et al. [8] trained the support vector machine for VTE risk prediction on cancer patients. However, methods from above studies are pre-designed and limited, which hardly take full advantages of different quantities of patient information in medical records. They are also limited in discovering new knowledge such as other potential variables associated with the disease. Recently deep learning (DL) technology has succeeded in analyzing medical images to diagnose skin cancer, detect pulmonary nodule and classify diabetic retinopathy [9–11] due to its advantages of capturing complex patterns in data, and some DL models [12, 13] were also proposed to combine medical ontologies to analyze high-dimensional and heterogeneous medical records but their results lack of interpretability. Although using attention mechanism in Retain [14] and GRAM [15] helps to explain features’ weights in low layer, the effect of features on the target disease remains unknown. In addition, training the DL model usually needs large sample size, which is infeasible for some diseases with low morbidity.

**Fig. 1:**
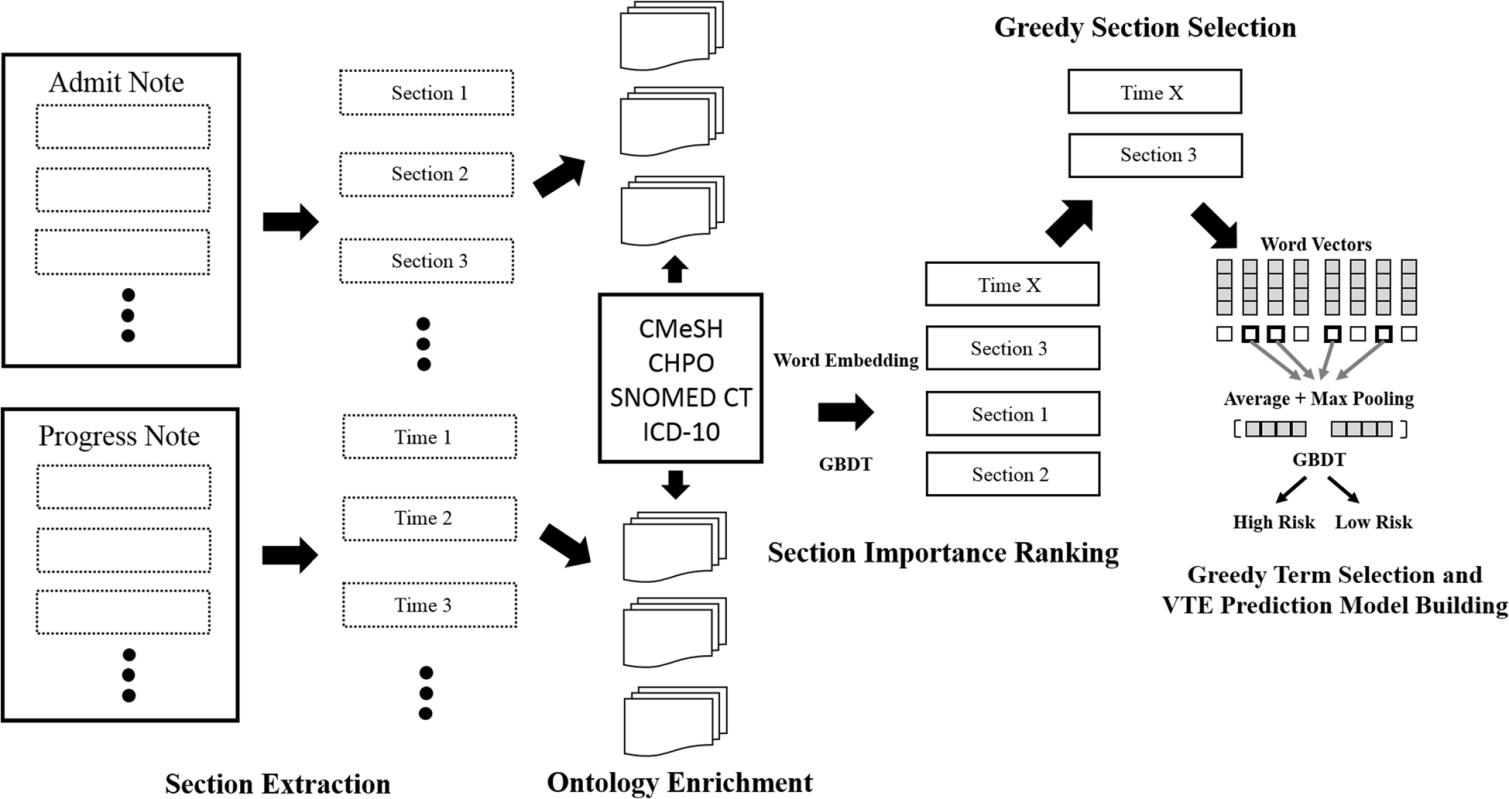
The workflow of VTE prediction model construction from medical records

In order to help the clinicians to explore new candidate VTE risk factors and develop efficient prediction model with certain interpretability from medical records, we propose an ontology-based approach which processes the free-text in medical records carefully, automatically evaluates importance of terms from ontologies and finally constructs the model based on candidate terms. The workflow of ontology extraction and risk assessment model establishment was summarized in Fig. 1. Interested sections in medical records are extracted automatically at first and then terms of ontologies are enriched according to their importance rankings within the text. Next significant text sections are selected by comparing their predictive validity using ML models. Finally, key terms are found by greedy section/term selections and VTE risk assessment model is built based on these terms. Results generated by the whole workflow can inspire further medical studies.

## Methods

### Medical records from Chinese hospital

In this study medical records of 3106 inpatients were collected from Peking Union Medical College Hospital (PUMCH) and each patient had two documents, admission note and progress note. Both documents were unstructured and had lots of paragraphs consisting of free text. The admission note usually included 11 sections: chief complaint, present history, previous history, personal history, family history, obstetrical history, menstrual history, physical examination, laboratory examination, admitting diagnosis and physician’s signature, and the progress note had daily description about the patient’s condition. A commonly situation in China [16] is that informatization systems which store medical records, medication and diagnosis codes are different and data cannot be shared and interchanged expediently in PUMCH. Hence only unstructured text information were downloaded and analyzed.

Inpatients were selected randomly based on their case number admitted to the same department in the internal medicine department of PUMCH from January 2014 to June 2016. Among them, 224 VTE inpatients were checked and Padua scores were calculated by the clinicians in previous study [5]. The inclusion criteria consisted of patients over 18 years old and patients with a hospital stay length ≥ 72 h. The exclusion criteria included patients receiving anticoagulation treatment (except for anticoagulation for the treatment of VTE diagnosed during hospitalization). DVT was diagnosed by venography or color Doppler ultrasonography. PE was diagnosed by pulmonary angiography, computed tomographic pulmonary arteriography, MRI or radionuclide lung ventilation-perfusion scans (V/Q scans).

### Ontology sources

In order to obtain comprehensive information of patients involving symptoms, diagnoses, drugs, operations and so on, multiple kinds of ontologies were gathered, including the Chinese versions of Medical subject headings (MeSH) [17], Human Phenotype Ontology (HPO) [18], SNOMED-CT [19] and ICD-10 [20]. The Chinese HPO was downloaded from the website (http://www.chinahpo.org) by applying to the Chinese Human Phenotype Ontology Consortium (CHPO). ICD-10 codes utilized in Chinese hospitals are translated and provided by National Health Commission of the People’s Republic of China. Chinese versions of SNOMED and Mesh (CMesh) were given by Institute of Medical Information/Medical Library, CAMS & PUMC. A translated version of SNOMED-CT by crowdsourcing approach was also included. After preprocessing and removing duplicates, 37,111 ICD codes, 11,903 phenotypic abnormalities from HPO, 55,750 MeSH words, 11,652 terms from SNOMED and 101,033 terms of crowdsourcing SNOMED-CT were kept and merged as the final ontology set with 156,918 unique terms.

### Rule-based section extraction

**Fig. 2:**
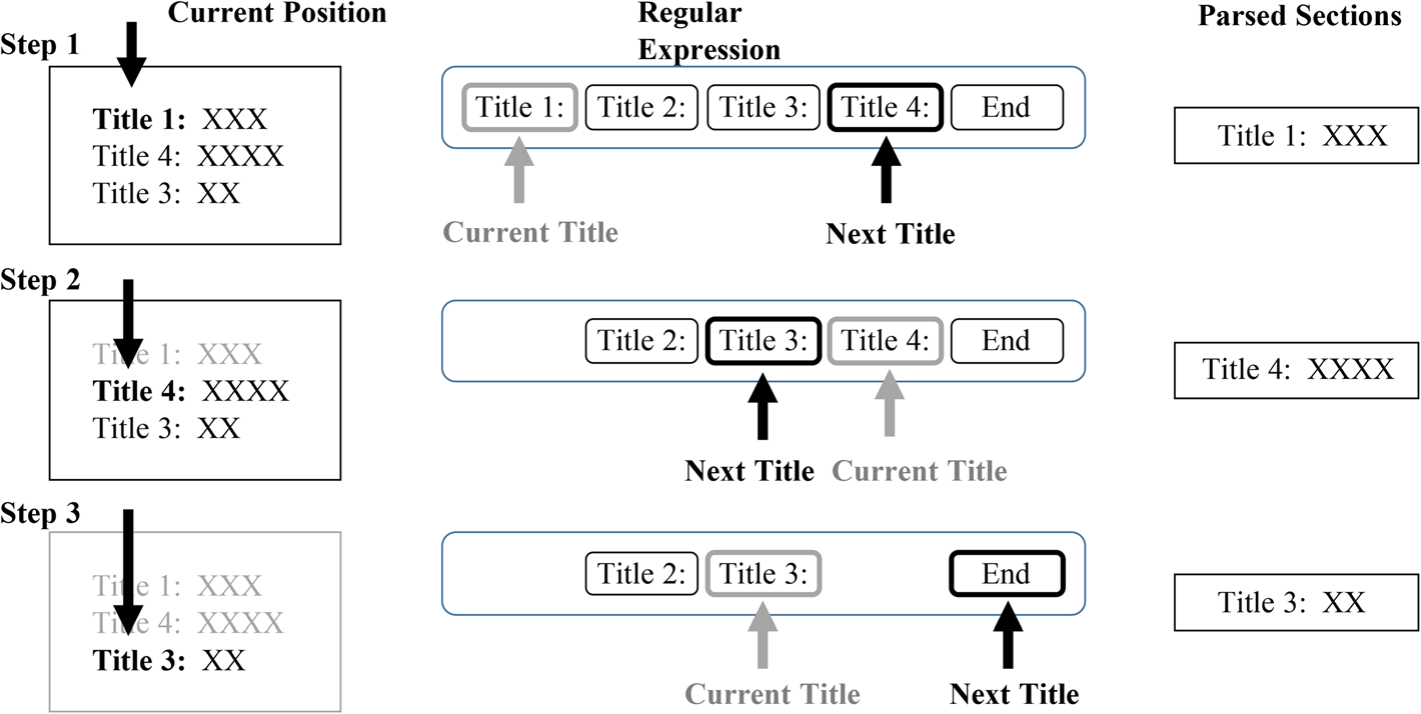
One example of the greedy section match algorithm

The first step of workflow was parsing sections within medical records. Due to the pattern that sections started with specific titles and white space between two sections, we utilized the regular expression to capture the start position of one section. Considering that some sections were removed by clinicians for convenience and orders of them could be changed, a greedy match algorithm was implemented to find the title of section having the minimum distance with current position iteratively. The text between two titles was regarded as a section related to the first title and current position in the document was updated after a section was recognized. One example of the algorithm was shown in Fig. 2. For the admission note, 8 sections were saved excluding the obstetrical history, menstrual history and physician’s signature. For the progress note, the daily objective descriptions were split by the date.

### Automatic ontology enrichment

With sections in medical records, we then searched for terms in ontologies that existed in different sections. Terms were sorted them according to their characteristics and weights in documents in order to shrink the size of term set. For each section, the term set was constructed respectively and each section had two groups of term sets based on non-VTE patients and VTE patients separately.

First sentence within a section was split via punctuation and candidate words were parsed using word segmentation tool, JIEBA (https://github.com/fxsjy/jieba), and then all sentences from medical records of VTE and non-VTE patients were combined into one file. The corpus was 106.7 Mb with about 1.3 million sentences. The universal distributed representations of words were inferred by the word2vector algorithm [21] provided by the GENSIM python package [22]. Words with frequencies less than 2 were omitted and the window size for predicted word was set to 5. The continuous Skip-gram model was used and finally each word was represented as a real number vector.

Terms which did not exist in candidate words were filtered and four features were calculated for the remaining terms,

1. *Word frequency F*_*word*_: ontology occurance times in specific section from all patients were counted and average frequency was given by dividing the total number of words in the section.
2. *Document frequency F*_*doc*_: The number of sections containing the ontology and the frequency was obtained by dividing the total number of the section in all patients. One notable observation was that sections with different dates in the progress note belonged to the same kind of section.
3. *Positive and negative times* (*N*_*pos*_ and *N*_*neg*_): when one term collocated with negative words such as ‘无 (not have)’, ‘未见 (not see)’, ‘没有 (no)’, ‘不像 (not like)’, ‘不似(not similar)’, ‘否认 (deny)’ and ‘不可能(not possible)’, it was regarded as one negative statement, otherwise it was a positive statement. Due to the fact that the sentence containing the term can be positive or negative statement, which reflected different contribution to the disease, positive and negative statements’ times of one term in the section were computed for every patient. Because objective expression about the patient usually owned simple phrasing, we used rule-based approaches and listed some common templates to judge the sentence. With times of positive and negative statements (*N*_*pos*_ and *N*_*neg*_) for the term, we could calculated the consistency of statements with the formula,

$$ {Consistency}_{word}=\frac{\mid {N}_{pos}-{N}_{neg}\mid }{\mathit{\max}\left({N}_{pos},{N}_{neg}\right)}. $$

When one term had most of positive or negative statements for one patient, its consistency was high. The mean value of *Consistency*_*word*_ for all VTE patients, denoted as $\overline{Consistency_{word}}=\frac{1}{N}{\sum}_{i=1}^N{Consistency}_{word}(i)$ where N was the number of patients and *Consistency*_*word*_(*i*) was the consistency of term in *i*^*th*^ patient, reflecting the term’s consistency of the VTE. High consistency of the term meant that its meaning was relatively definite and stable. In addition, the variation of consistency of one term among patients implied attention from the clinician because terms existed in some text templates usually.

had low variation, and high standard deviation of consistency,

$$ \upsigma \left({Consistency}_{word}\right)=\sqrt{\frac{\sum_{i=1}^N{\left({Consistency}_{word}(i)-\overline{Consistency_{word}}\right)}^2}{N-1}}, $$

showing that the term attracted more discussions in medical records.

**Fig. 3:**
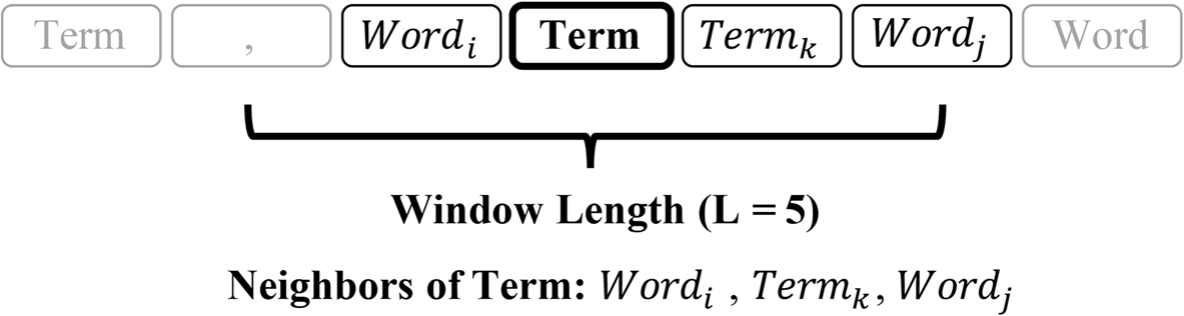
Neighbors of one term when calculating first order neighbors entropy

4) *Entropy of first order neighbors S*_*neighbor*_: Some words might be common in various phrases such as some terms about human body and their neighbors could be diverse, resulting in low ability to describe the disease. So neighbors of one term were taken into account. Words that belonged to the same sentence without the segmentation of punctuation and located in the window centered around the term were numerated (See Fig. 3) and the entropy was estimated below,

$$ {S}_{neighbor}=-\sum \limits_k^K{P}_k\mathit{\ln}{P}_k $$

where K was the number of unique neighbors of interested term within window with length L and $$ {P}_k=\frac{N_k}{\sum \limits_i{N}_i} $$ was frequency of word k within the window. *N*_*k*_ was total counts of word k when its distance with the term was not more than L. To penalize common terms existed in many terminologies, we define the penalty term as $$ {Penalty}_{neighbor}=\raisebox{1ex}{$1$}\!\left/ \!\raisebox{-1ex}{$1+{S}_{neighbor}$}\right. $$ . When one term’s neighbors were diverse, its *S*_*neighbor*_ was relatively large and $$ \raisebox{1ex}{$1$}\!\left/ \!\raisebox{-1ex}{$1+{S}_{neighbor}$}\right. $$ became small.

Then the importance of one term to the section was computed by the following formula,

$$ {W}_o={F}_{word}\times {\mathit{\log}}_{10}\left(\frac{1}{F_{doc}}\right)\times {Penalty}_{neighbor}\times \overline{Consistency_{word}}\times \left(1+\upsigma \left({Consistency}_{word}\right)\right), $$

where ${F}_{word}\times {\mathit{\log}}_{10}\left(\frac{1}{F_{doc}}\right)$ was the frequency-inverse document frequency (TF-IDF) [23]. For every section, terms were sorted in descending order with their weights and top K terms were saved for further analysis.

### Section evaluation by ML methods

The importance of terms to VTE risk assessment was evaluated in unit of the section at first. Features of specific section were constructed based on terms within this section and four ML methods including random forest (RF) [24], gradient boosting decision tree (GBDT) [25], logistic regression (LR), and support vector machine (SVM) [26] were built to compare AUC scores among different sections. For each section, word vectors of terms generated in the ‘*automatic ontology enrichment*’ step were added together and averaged as follows,

$$ {V}_{patient}=\frac{1}{K}{\sum}_{j=1}^K{V}_j $$

where K was the number of terms in the section and *V*_*j*_ ∈ *ℝ*^*P*^ represented a word embedding with P dimensions for term j. In addition, max pooling operation was adopted for every dimension of the word embedding,

$$ {M}_{patient}=\mathit{\operatorname{Max}}- pooling\left({V}_1,{V}_2,\dots, {V}_K\right) $$

where *M*_*patient*, *j*_ was the maximum value of the set {*V*_1, *j*_, *V*_2, *j*_, …, *V*_*K*, *j*_} and *V*_1, *j*_ meant the *j*^*th*^ element of vector *V*_1_. Then one patient was represented by concatenating two vectors together [27],

$$ {X}_{patient}=\left[{V}_{patient}\kern0.5em {M}_{patient}\right]. $$

Two kinds of AUC scores were estimated, AUC (Only) and AUC (Exclusion). The former was calculated based on models using features from the current section only, while the latter was based on models utilizing features from all sections except the current. When combining multiple sections, word embeddings of terms in these sections were averaged and performed max-pooling operation together. The AUC (Only) reflected the direct importance of terms belonging to some section and the AUC (Exclusion) showed the terms’ relative effect on the VTE prediction. Finally sections were ranked according to their AUC (Only) and the order reflected the importance of the section to VTE risk assessment.

During the model training process, 20% non-VTE and VTE inpatients were chosen randomly as test set and the remaining 80% was utilized to train ML model. 5-fold cross validation were done to compute the mean and standard deviation of AUC scores.

### Ontology-based VTE risk assessment model

With the importance ranking of terms and sections, we proposed an approach to automatically select the section and pick terms associated with VTE from selected sections to build VTE risk assessment model. This approach consisted of two steps: choose the best section set using all terms in sections via a greedy selection process and next, find the optimal term set from terms of the section set to build GBDT model.

**Fig. 4:**
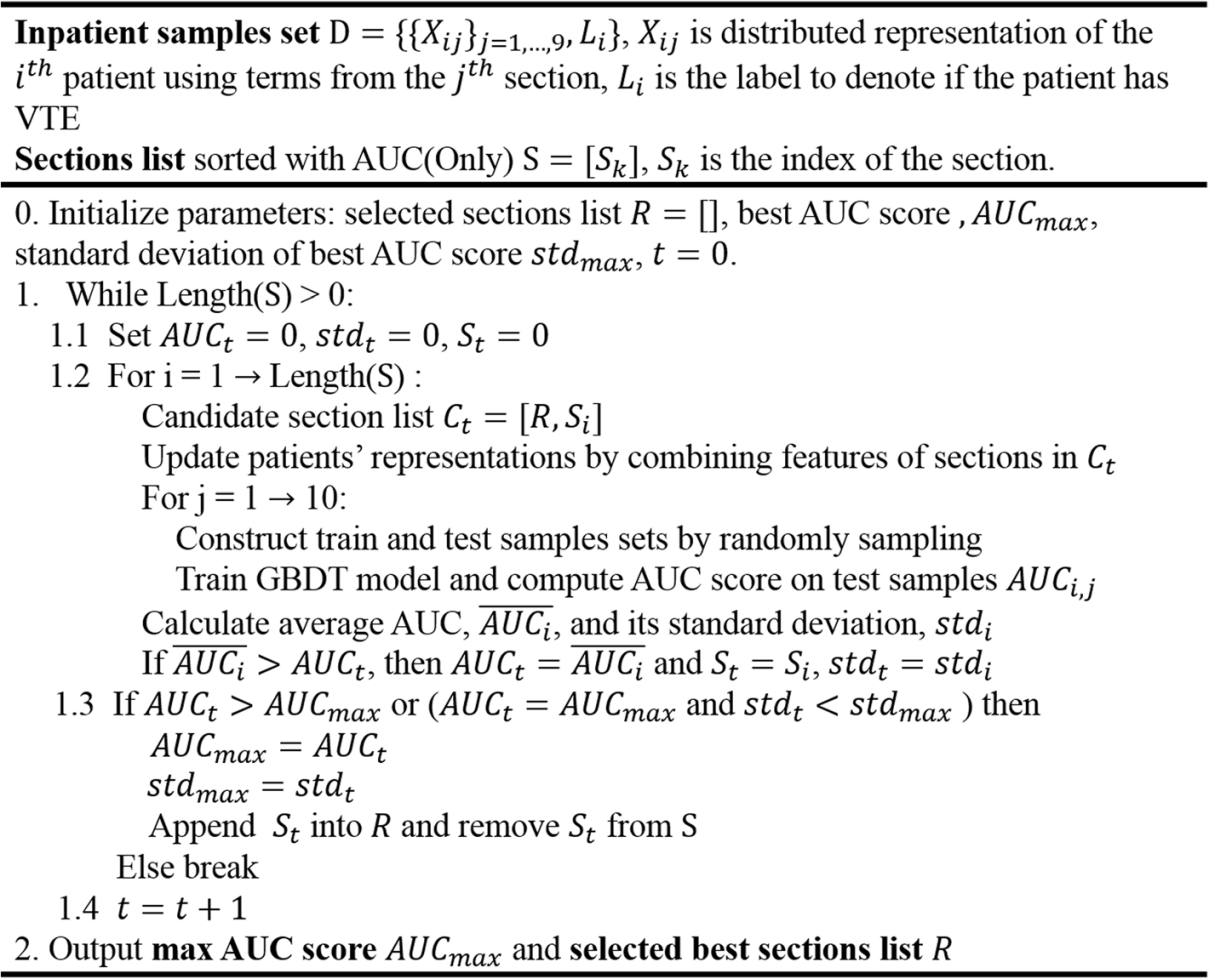
Section evaluation process of VTE risk assessment model

Firstly, just like the greedy feature selection method [28], in each iteration, all terms from one section with relatively high AUC (Only) were added to construct new features and GBDT models were re-trained to try to improve current prediction performance. Patients’ distributed representations were updated by re-calculating average values of word vectors and max-pooling vectors of terms. During each round the section which obtained higher AUC improvement than other sections was then added into the section set. The selection process stopped when there was no increase in mean values or standard deviation of AUC scores. We explained the section evaluation process in details at Fig. 4.

Given the section set with the highest prediction validity, we try to find best subset of terms of among the sections to build VTE prediction model. Similar with the greedy construction of section set, instead of testing per section, we try to add each term consecutively into the term set to evaluate its performance in each round. The term with relatively higher mean AUC value was put into the term set preferentially. In order to better assess the relationship between the number of terms and AUC scores, this process stopped until the term set contained all terms. Then the optimal number of terms and term subset was obtained by fitting a curve and searching for the maximum of mean AUC scores. Considering the fact that number of terms is much larger than sections’ number, parallel computing was utilized to reduce running time.

## Results

### Demographic characteristics of inpatients

Demographic and clinical features of VTE and non-VTE inpatients were shown in Table 2. Compared with 2,882 non-VTEs, the gender ratio 1:1.13 and mean BMI value 23.99 ± 4.13 Kg/m^2^ of VTE patients showed no significant difference. VTEs’ average age 55.81 ± 16.45 and mean hospital day 22 (14, 35) were both obviously higher than the non-VTE (*P* value < 0.05). In addition, Padua model’s results and values of its 11 risk factors were also calculated. There was notable difference in Padua score (5.88 ± 2.46 v.s. 2.89 ± 2.45) as well as the ratio of high risk patients (86.61% v.s 37.40%) between the two groups, of which values were higher within VTE patients. Among 11 risk factors, ratios of active malignant cancer/chemotherapy (31.25%), recent trauma/surgery (6.25%), acute myocardial infarction/ischemic stroke (3.57%), and BMI > =30 kg/m^2^ (5.80%) for VTE patients were not significantly different from patients without VTE. However, ratios of remaining seven factors of the VTE were higher than the non-VTE. Reduced mobility (70.54%), ongoing glucocorticoid treatment (60.71%), acute infection/rheumatologic disorder (58.48%), heart/respiratory failure (29.12%) were the most common risk factors for VTE inpatients. The significance test was done as follows. Comparison between the two groups for risk factor that did not obey the normal distribution was done with the Mann-Whitney U test, and two-sided independent student t-test was used to compare the other variables’ significance, with a threshold of 0.05.

**Table 2:** Demographic characteristics of inpatients

	VTE	non-VTE	P value	Total
N	224	2882	–	3106
Gender (Male)	119 (53.13%)	1587 (55.07%)	> 0.05	1673
Age (Year)	55.81 ± 16.45	52.75 ± 16.24	< 0.05	52.97 ± 16.28
BMI (Kg/m^2^)	23.99 ± 4.13	23.47 ± 4.27	> 0.05	23.51 ± 4.26
Hospital stay (Day)	22 (14, 35)	11 (6, 18)	< 0.05	12 (6, 19)
Padua Score	5.88 ± 2.46	2.89 ± 2.45	< 0.05	3.10 ± 2.57
High Risk (Padua model)	194 (86.61%)	1078 (37.40%)	< 0.05	1272
Active malignant cancer/chemotherapy	70 (31.25%)	807 (28.00%)	> 0.05	877
Previous VTE	34 (15.28%)	17 (0.59%)	< 0.05	51
Reduced mobility	158 (70.54%)	1030 (35.74%)	< 0.05	1188
Thrombophilic condition	25 (15.63%)	53 (1.84%)	< 0.05	88
Recent trauma/surgery	14 (6.25%)	87 (3.02%)	> 0.05	101
Age > =70	45 (20.09%)	418 (14.50%)	< 0.05	463
Heart/respiratory failure	65 (29.12%)	112 (3.89%)	< 0.05	177
Acute myocardial infarction/ischemic stroke	8 (3.57%)	44 (1.53%)	> 0.05	52
Acute Infection/rheumatologic disorder	131 (58.48%)	710 (24.64%)	< 0.05	841
BMI > =30 kg/m^2^	13 (5.80%)	165 (5.73%)	> 0.05	178
Ongoing glucocorticoid treatment	136 (60.71%)	972 (33.73%)	< 0.05	1108

Hospital stay is denoted as ‘Median (lower quartile, upper quartile)’. Age, BMI and Padua score was expressed with ‘Mean ± Standard Deviation’

### Terms of ontologies from nine kinds of sections

Basic information of terms within distinct sections from non-VTE and VTE patients respectively during the process of ‘*Automatic Ontology Enrichment*’ were shown in Table 3, and the number of terms and neighbors were listed. It can be seen that the section ‘Present History’ had the most terms (1,162 in non-VTE and 1,244 in VTE) and the ‘Personal History’ had the least (20 in non-VTE and 18 in VTE) in both non-VTE and VTE. As for the neighborhood information, size of neighbors of terms in the ‘Progress Note’ was much larger more than other sections (148 in non-VTE and 31 in VTE), and conversely, the ‘Chief Complaint’ had the smallest neighbors size (13 in non-VTE and 2 in VTE). Generally counts of terms between the non-VTE and the VTE were comparable but terms in the non-VTE had more abundant neighbors, which may be resulted from their gap in the number of samples.

**Table 3:** Number of terms of ontologies within different sections

Section Name	Non-VTE		VTE	
	Term	Neighbor	Term	Neighbor
Chief Complaint	44	13 (6, 28)	53	2 (1, 4)
Present History	1162	37 (22, 75)	1244	4 (2, 6)
Previous History	197	32 (18, 53)	224	4 (2, 6)
Personal History	20	16 (9, 32)	18	3 (2, 5)
Family History	28	22 (9, 33)	26	4 (1, 6)
Physical Examination	391	16 (7, 30)	380	3 (2, 6)
Laboratory Examination	385	29 (18, 49)	344	3 (2, 6)
Admitting Diagnosis	175	42 (29, 67)	211	5 (3, 8)
Progress Note	733	148 (92, 267)	811	31 (17, 58)

The ‘Neighbor’ is the number of distinct words around terms with a window length 5 and is expressed with ‘Median (lower quartile, upper quartile)’

During the process of automatic ontology enrichment by sorting terms according to their importance, results obtained via adding information of entropy of neighbors and consistency of terms differed from the order that uses TF-IDF values only. For example, in the section ‘Progress Note’, terms ‘左侧 (left side)’, ‘可能 (maybe)’, ‘右侧 (right side)’ and ‘继续 (continue)’ were among top 10 terms calculating by TF-IDF, which had abundant neighbors but low standard deviation of term consistency. Our proposed scoring approach penalized these terms and revealed more professional terms in the ranking of terms, such as ‘血细胞 (hemocyte)’, ‘呼吸音 (breath sounds)’, and ‘呼吸 (breathe)’, of which their TF-IDF values were low.

### Comparison of section importance on VTE risk prediction

**Fig. 5:**
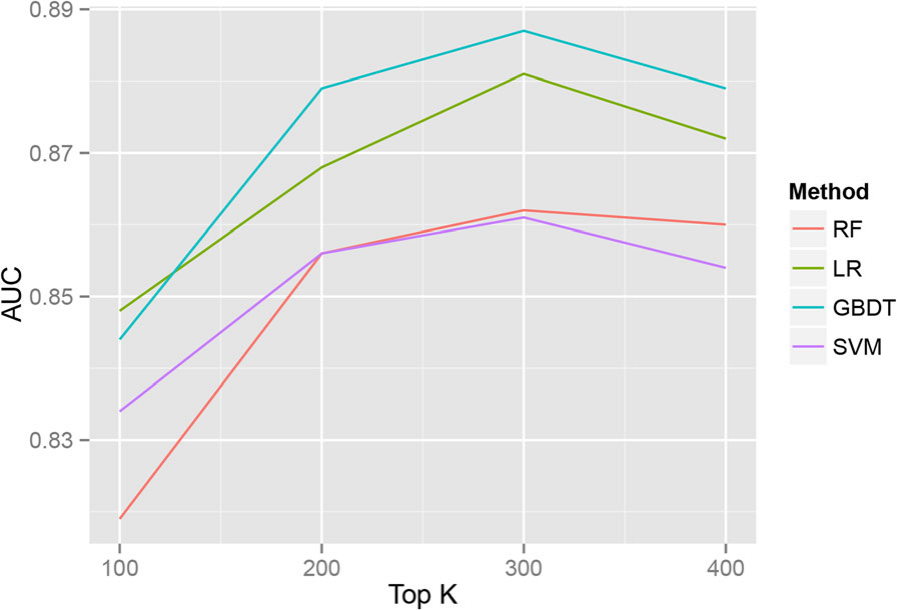
AUC scores of four ML methods using top K = 100, 200, 300 and 400 terms

To decide the number of terms in the section we should choose and the best suitable ML method, we compared four ML models’ AUC scores by selecting top K = 100, 200, 300, 400 terms in each section respectively when fixing word embedding dimension (*P* = 20). Terms of all sections were combined to build patients’ distributed representations and results could be found in Fig. 5**.** It could be seen that four ML methods achieved the highest AUC values using top 300 terms in every section and GBDT performed better than other methods. So for the following experiments, we extracted top 300 terms of each section from VTE patients. Then the optimal word embedding dimension and the most suitable ML method were determined by comparing AUC scores of four ML approaches when *P* = 10, 20, 40, 80, 100, 120, 150, and 200. It could be seen that the performance of GBDT was better than other methods on most of conditions, and when the dimension was more than 100, AUC scores of GBDT tended to be stable (Table 4). Therefore the most appropriate dimension of word vector was *P* = 100 and GBDT approach was chosen as default.

**Table 4:** Comparison of AUC scores of four ML models using word vectors with different dimensions

Dimension	10	20	40	80	100	120	150	200
AUC (GBDT)	0.863 ± 0.024	0.891 ± 0.023	0.916 ± 0.026	0.916 ± 0.017	0.929 ± 0.014	0.929 ± 0.015	0.927 ± 0.020	0.927 ± 0.020
AUC (RF)	0.852 ± 0.022	0.871 ± 0.030	0.883 ± 0.023	0.881 ± 0.029	0.884 ± 0.021	0.893 ± 0.020	0.897 ± 0.018	0.884 ± 0.019
AUC (LR)	0.851 ± 0.023	0.884 ± 0.019	0.912 ± 0.019	0.921 ± 0.020	0.926 ± 0.022	0.923 ± 0.022	0.920 ± 0.026	0.923 ± 0.014
AUC (SVM)	0.862 ± 0.019	0.873 ± 0.034	0.861 ± 0.031	0.879 ± 0.021	0.869 ± 0.025	0.880 ± 0.031	0.867 ± 0.023	0.830 ± 0.034

The value of AUC score is formatted with ‘Mean value ± Standard deviation’. All terms are used to train models

AUC (Only) and AUC (Exclusion) scores of 9 types of sections were shown at Table 5. Obviously terms from the ‘Progress Note’ had the highest AUC (Only) score, 0.939 and results excluding them led to the worst AUC (Exclusion) value obviously, 0.784, verifying its key role in VTE prediction. Apart from the ‘Progress Note’, AUC (Exclusion) scores of remaining sections were similar, which implied that efficiency of terms of them weren’t of much difference. Only considering the AUC (Only), the second best section was the ‘Admitting Diagnosis’ with the value 0.754 and terms from the ‘Personal History’ showed the lowest score, 0.564. One interesting observation was that when we used terms in all sections, the prediction validity (0.929) was less than the result from a single section such as the ‘Progress Note’, which reflected the importance of ontology and section evaluation. Comparing the values of AUC (Exclusion) of sections and AUC (All) using all sections, removing terms from ‘Previous History’ and ‘Physical Examination’ increased AUC (All). A possible reason could be that terms from the two sections introduced more noises than useful information for model training.

**Table 5:** AUC scores of GBDT models using only or excluding some section

Section Name	AUC (Only)	AUC (Exclusion)
Chief Complaint	0.635 ± 0.029	0.930 ± 0.015
Present History	0.748 ± 0.023	0.933 ± 0.021
Previous History	0.610 ± 0.033	0.927 ± 0.019
Personal History	0.564 ± 0.019	0.929 ± 0.017
Family History	0.605 ± 0.029	0.926 ± 0.023
Physical Examination	0.711 ± 0.019	0.928 ± 0.021
Laboratory Examination	0.638 ± 0.036	0.940 ± 0.016
Admitting Diagnosis	0.754 ± 0.034	0.923 ± 0.017
Progress Note	0.939 ± 0.018	0.784 ± 0.035
ALL	0.929 ± 0.014	

The value of AUC score is formatted with ‘Mean value ± Standard deviation’. AUC scores with one specific section are denoted as ‘AUC (Only)’ and results excluding some section are ‘AUC (Exclusion)’. The ‘ALL’ means that terms from all sections are used

### Best section set on VTE prediction

**Fig. 6:**
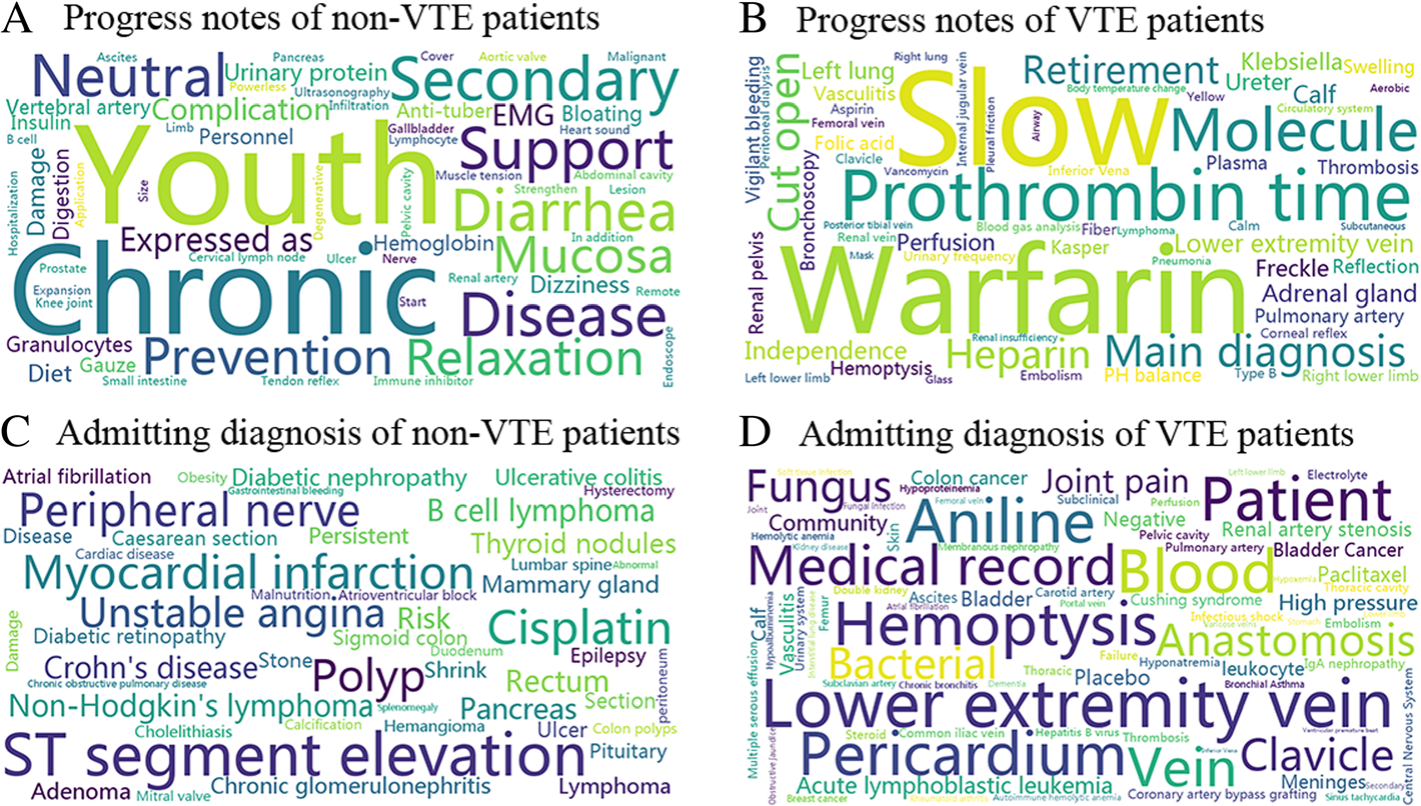
Unique terms within progress notes and admitting diagnosis only from VTE or non-VTE patients

By selecting terms and sections greedily, terms of two sections, namely ‘Progress Note’ and the ‘Admitting Diagnosis’, were chosen and achieved the best VTE assessment performance. Using all terms from these two sections, the best mean AUC values, 0.951 ± 0.009, was obtained which were higher than the traditional Padua model (0.803 ± 0.027). Furthermore GBDT model’s sensitivity (0.877 ± 0.019) was similar with the Padua (0.897 ± 0.046) while specificity of GBDT (0.880 ± 0.006) showed obvious superiority over the latter (0.617 ± 0.015). Because terms used by GBDT models were ranked only based on medical records of VTE patients, we visualized unique terms existed only in non-VTE’s or VTE’s ‘Admitting Diagnosis’ and ‘Progress Note’. Results were plotted at Fig. 6 by the word cloud toolkit (https://github.com/amueller/word_cloud) for ease of comparison.

There were 237 common terms in ‘Progress Note’ and 126 identical terms in ‘Admitting Diagnosis’ between VTE and non-VTE. Medical records of VTE patients had 63 and 85 unique terms in ‘Progress Note’ and ‘Admitting Diagnosis’ respectively. Non-VTE patients had 63 distinct terms in ‘Progress Note’ and 49 particular terms in ‘Admitting Diagnosis’. Visual differences of terms between non-VTE and VTE can be found. Among the terms of VTE patients’ progress notes, ‘warfarin’, ‘prothrombin time’, ‘heparin’ and ‘retirement’ were ranked higher in the list, which were associated with the disease. In the admitting diagnosis, VTE related terms such as ‘veins of lower extremity’, ‘aniline’ and ‘hemoptysis’ were within the top five. However, some terms which might be meaningless were also included such as ‘bluntness’, ‘molecule’ and ‘patient’, which should be filtered in the following process of model deviation.

### Performance of new VTE risk assessment model

**Fig. 7:**
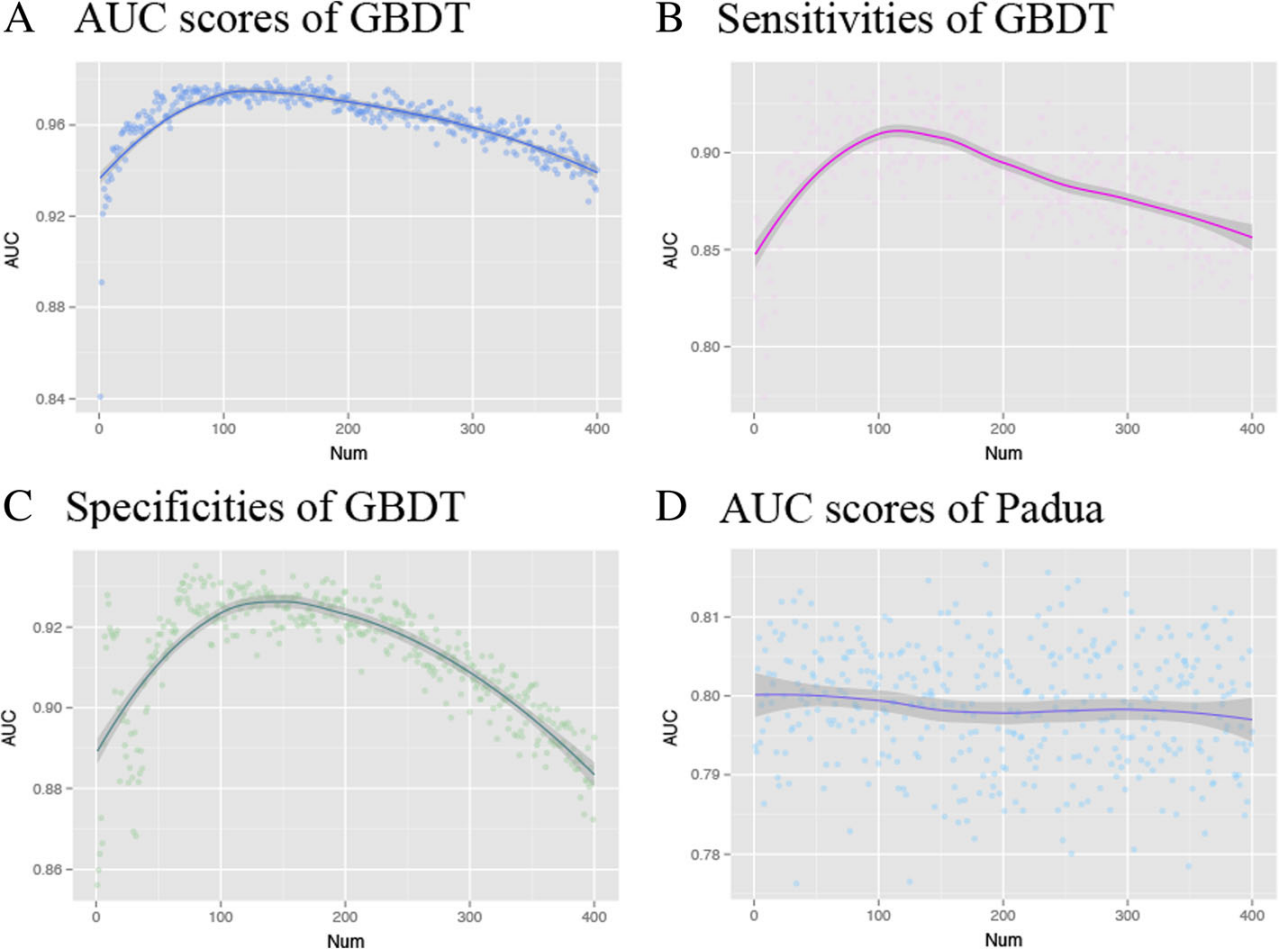
Relationships between predictive validity of two models, GBDT and Padua, and the number of terms

Based on terms from two sections, ‘Progress Note’ and ‘Admitting Diagnosis’, results of greedy term selection were plotted in Fig. 7. Figure 7a showed how AUC scores of GBDT model changed with the number of added terms and model’s performance achieved the optimal AUC (0.973 ± 0.006) using about 110 terms. Changing curves of sensitivities and specificities of GBDT were also shown at Fig. **7**b and c, and the best model’s sensitivity (0.900 ± 0.037) and specificity (0.918 ± 0.012) with 110 terms were nearby the maximum. Conversely, the performance of Padua model was stable (Fig. 7d) and its AUC values were fluctuating around 0.80. ML model utilizing 110 terms had higher predictive validity than the Padua (AUC: 0.791 ± 0.022, Sensitivity: 0.846 ± 0.049, Specificity: 0.628 ± 0.013).

We further organized the top 110 terms into several groups based on their relations with VTE events, and some typical terms were listed at Table 6. Terms in the medical record included the following two aspects. First, terms concerning diagnostic terminologies and susceptible sites included ‘embolism’, ‘veins in the lower limbs’, ‘left lower limb’ and ‘shrank’, which were obviously related to the VTE events. Secondly, some terms representing risk factors of VTE were also found in the medical record. For instance, as a VTE risk factor in consensus, tumor was expressed with keywords of ‘lymphoma’, ‘paclitaxel’, ‘adenocarcinoma’. Similarly, surgery and invasive operation were illustrated in terms of ‘resection’, ‘cholecystectomy’ and ‘stenting’. Furthermore, rheumatic immune disease and acute infection indicated by terms of ‘rheumatoid arthritis’, ‘lupus nephritis’ and ‘septic shock’ were also in accordance with the VTE events.

**Table 6:** Classification of typical terms proposed by ML model

Classification Name	Typical Terms
Predilection site and clinical manifestation	Thrombus, Lower limb vein, Left lower limb, Posterior tibial vein, Thrombosis, Femoral vein, Embolism, Shank, Hemoptysis, Pulmonary artery
Treatment	Warfarin
Tumor	Paclitaxel, Lymphoma, Lung adenocarcinoma
Surgery, Trauma, Invasive Operation	Resection, Peritoneal dialysis, Cholecystectomy, Stenting
Rheumatic Disease	Rheumatoid arthritis, Prednisone, Hyperuricemia, Lupus nephritis
Acute Infection	Bacteria, Pneumonia, Antibiotics, Septic shock, Vancomycin, Soft tissue infection
Mechanical Ventilation	Mask

## Discussion

Proposed ontology-based workflow not only evaluates the importance of terms within various sections of medical records, but also builds efficient new risk prediction model, with the assistance of NLP and ML technologies. Terms used for building VTE prediction model can help clinicians explore disease’ risk factors more conveniently. The efficiency of the workflow was verified on medical records from PUMCH.

In general, there are plenty of words in medical free text unrelated to the disease and different sections of medical records usually have distinct aspects about patients information. Based on these facts multiple types of medical ontologies are utilized and terms in ontologies are classified according to different types of sections. From Table 3, the differences of terms’ counts and the number of neighbors among sections are apparent. Further terms’ number within sections is not directly correlated with the section’s disease prediction ability combining with Table 5. Although terms in the ‘Present History’, ‘Physical Examination’ and ‘Laboratory Examination’ are more abundant than the ‘Admitting Diagnosis’, AUC (Only) scores of the former are relatively lower. In addition, by comparing average positions of 110 terms proposed by new VTE risk assessment model in two terms lists ranked by our ontology enrichment method and traditional TF-IDF respectively (Table 7), the average position of ontology enrichment is relatively better than the TF-IDF, reflecting the superiority of ontology enrichment again.

**Table 7:** Terms’ average positions of ontology enrichment and TF-IDF methods

Top@K	10	30	50	70	90	110
Ontology Enrichment	146.6	214.1	218.9	218.7	215.6	218.7
TF-IDF	161.4	238.0	248.4	242.9	237.2	235.8

The ‘Top@K’ means top K terms among 110 terms proposed by new VTE risk assessment model

One notable thing is that combining comprehensive terms don’t get the best prediction performance. Results from Table 5 and Fig. 7 show that choosing sections associated with the disease and using subset of terms can obtain more promotion on VTE risk assessment. Although collecting numerous data about patients becomes a reality and while many popular end to end methods propose to mining patients’ patterns directly from raw data [12, 29], we argue that identifying significant information and removing noises are still very important. By ontology selection and removing redundant sections we may improve model’s efficiency further. In Table 6, terms proposed by the model include not only diseases and medications but also body parts, symptoms and common expressions. Current DL methods [30, 31] usually utilize structural information and only consider limited types of codes about diseases, drugs and procedures to build the model. These are not enough to describe interested diseases of interest. Multiple types of ontologies and unstructured clinical notes should be taken into account.

The final VTE risk assessment model based on picked terms show higher sensitivity, specificity and AUC than the Padua. Although the ML model seems to perform better, its differences with the Padua should be emphasized. In reality the ML model used much more features, 110 terms, compared to the Padua with 11 factors. Padua model uses medical knowledge, which is summarized from clinical practice, instead of words written by clinicians. Just checking existence of specific terms may result in overfitting or inferior generalization. In future, classification of terms and utilizing structures of ontologies, e.g. the knowledge graph, to associate terms in clinical notes with medical knowledge are necessary, providing better interpretability. Considering that only one hospital’s clinical dataset was used to train VTE risk assessment model for Chinese inpatients, multiple-center research is needed to evaluate its generalizability further. In addition, small sample size of VTE patients is also a limitation. More medical records should be collected and new approaches to model derivation based on ontologies need to be explored.

Since VTE is a complex multi-factor disease, the effect of risk stratification with limited factors based on the existing experience is not satisfactory (studies shown that the Padua model has poor performance and low specificity). The ML model developed in this study can help clinicians to judge the risk stratification of patients more accurately, as well as find out new risk factors and potential VTE patients ignored by the physicians. Meanwhile, risk prediction using the keywords in the medical record text through the electronic medical record system, is simpler and much more convenient, which provides novel ideas for discovering new disease mechanisms.

## Conclusions

In this study, a method of ontology-based VTE risk factors mining and model establishment from medical records is validated on real clinical dataset from PUMCH. Selected terms and sections from medical records help the clinicians discover potential VTE risk factors and GBDT model built based on top 110 terms improves the performance of VTE prediction. This method is expected to be applied in more diseases and embedded into the EHR system to assist clinical work.

## Abbreviations

AUC: Area under curve
DL: Deep learning
DVT: Deep venous thrombosis
EHR: Electronic health record
GBDT: Gradient boosting decision tree
HPO: Human phenotype ontology
LR: Logistic regression
Mesh: Medical subject headings
ML: Machine learning
NLP: Natural language processing
PE: Pulmonary embolism
PUMCH: Peking Union Medical College Hospital
RF: Random forest
ROC: Receiver operating characteristic curve
SVM: Support vector machine
VTE: Venous thromboembolism
